# Hepatitis E Virus Produced from Cell Culture Has a Lipid Envelope

**DOI:** 10.1371/journal.pone.0132503

**Published:** 2015-07-10

**Authors:** Ying Qi, Feng Zhang, Li Zhang, Tim J. Harrison, Weijin Huang, Chenyan Zhao, Wei Kong, Chunlai Jiang, Youchun Wang

**Affiliations:** 1 National Engineering Laboratory for AIDS Vaccine, Jilin University, Changchun, 130012, China; 2 Division of HIV/AIDS and Sexually-Transmitted Virus Vaccines, National Institutes for Food and Drug Control, Beijing, 100050, China; 3 Division of Medicine, University College London Medical School, London, WC1E 6BT, United Kingdom; Virginia Polytechnic Institute and State University, UNITED STATES

## Abstract

The absence of a productive cell culture system hampered detailed analysis of the structure and protein composition of the hepatitis E virion. In this study, hepatitis E virus from a robust HEV cell culture system and from the feces of infected monkeys at the peak of virus excretion was purified by ultra-centrifugation. The common feature of the two samples after ultracentrifugation was that the ORF2 protein mainly remained in the top fractions. The ORF2 protein from cell culture system was glycosylated, with an apparent molecular weight of 88 kDa, and was not infectious in PLC/PRF/5 cells. The ORF2 protein in this fraction can bind to and protect HEV RNA from digestion by RNase A. The RNA-ORF2 product has a similar sedimentation coefficient to the virus from feces. The viral RNA in the cell culture supernatant was mainly in the fraction of 1.15g/cm^3^ but that from the feces was mainly in the fraction of 1.21 g/cm^3^. Both were infectious in PLC/PRF/5 cells. And the fraction in the middle of the gradient (1.06g/cm^3^) from the cell culture supernatant,but not that from the feces, also has ORF2 protein and HEV RNA but was not infectious in PLC/PRF/5.The infectious RNA-rich fraction from the cell culture contained ORF3 protein and lipid but the corresponding fraction from feces had no lipid and little ORF3 protein. The lipid on the surface of the virus has no effect on its binding to cells but the ORF3 protein interferes with binding. The result suggests that most of the secreted ORF2 protein is not associated with HEV RNA and that hepatitis E virus produced in cell culture differs in structure from the virus found in feces in that it has a lipid envelope.

## Introduction

Hepatitis E virus (HEV) has long been known as a major cause of acute viral hepatitis, with an overall fatality rate of about 2% [1]. HEV is a small non-enveloped RNA virus with a size of 27–34 nm and is classified as a *Hepevirus* in the family *Hepeviridae*. There are four recognized genotypes of HEV that infect humans: genotypes 1 and 2 are thought to infect humans and non-human primates exclusively, whereas genotypes 3 and 4 seem to be of porcine origin [2]. The HEV genome is a linear, single-stranded, positive-sense RNA containing three open reading frames (ORFs): ORF1, the largest ORF, is believed to encode the viral nonstructural proteins [3]. ORF2 and ORF3 overlap and their proteins are translated from a single bicistronic subgenomic RNA [4]. ORF2 encodes the capsid protein and ORF3 encodes a small protein. However, in the absence of a productive cell culture system, a detailed analysis of the hepatitis E virion structure is lacking. HEV can be produced in cell culture using infectious cDNA clones and the ORF3 protein was found to be responsible for virion egress [5–8]. HEV proteins have been studied principally following high level expression using plasmid vectors. Although the full-length ORF2 protein consists of 660 amino acids, its apparent susceptibility to proteolytic cleavage means that its size in the virion is not known. The size of the ORF2 protein varies from 52kDa to 84kDa when it is over-expressed in insect or mammalian cells and its size in T = 1 or T = 3 crystal structures of HEV virus-like particles (VLPs) is not full length[9–12].

On the other hand, HEV infection is transmitted mainly via the fecal-oral route through virus contaminated water or food, although it also can be transmitted via transfusion.[2, 13]. Studies by Takahashi et al. and Emerson et al.[7, 14]have shown that the density of virions in serum and cell culture supernatants is lower than that of virions in feces but increases after delipidation. This suggests that virions in the circulation of infected individuals and those produced in cell culture are enveloped. The ORF2 protein binds RNA and forms the viral capsid and the ORF3 protein masks the ORF2 protein and the lipid envelopes the virion. However, whether the ORF3 is embedded in the lipid envelope is not known [8, 14].

In this study, we investigated hepatitis E virions produced from a robust cell culture system and purified from the feces of monkeys at the peak of virus excretion. The features of the virion and the ORF2 protein in ultra-centrifuge gradient fractions were studied with the aim of analyzing the composition of the virion from various sources.

## Materials and Methods

### Ethics Statement

All animal care, surgical, and research procedures are consistent with the Guide for the Care and Use of Laboratory Animals and were approved by the Institutional Animal Care and Use Committee of National Institutes for Food and Drug Control. Monkey was individually housed in stainless steel cages (L×W×H: 800×700×750 mm) under condition of 16–26°C, 40–70% relative humidity, a 12hr light-dark cycle, and a room air exchange of 8–10 times per hour. Meat mirrors were equipped in the cages as animal toys. The monkey was provided with 300 g of standard diet and fruits per day. Sterilized tap water was available ad libitum throughout the study.

#### 1. Virus in cell supernatants and virus in feces

Virus in cell culture supernatant:PLC/PRF/5 (ATCCCRL-8024) cells were grown in minimum essential medium (MEM; Hyclone, Logan, UT), supplemented with 10% (v/v) heat-inactivated fetal bovine serum (FBS), 100 U penicillin G ml^-1^ and 100 μg streptomycin ml^-1^, at 37°C in a humidified 5% CO2 atmosphere. Hepatitis E virus (virus accession: AJ272108, genotype:4, titer of the stock: 2.54×10^6^ copies/ml [15]) replicated efficiently in PLC/PRF/5 cells and the culture supernatants were collected periodically, concentrated by ultrafiltration and used for ultracentrifugation.

Virus in feces: feces from an acutely infected monkey at the peak of virus excretion (virus accession: JQ655736, genotype:4, titer of the stock: 2.58×10^6^ copies/ml[16]) were suspended in 10% phosphate buffer (PBS) (w/v) and filtered using 0.45μm membranes (Pall, Washington, NY). Then, the suspensions were used for ultracentrifugation.

#### 2. The quantification of HEV ORF2 protein and HEV RNA

The ORF2 protein expressed and purified from sf9 cells was diluted serially to 30ng/ml, 20 ng/ml, 10 ng/ml, 5 ng/ml, 4 ng/ml, and 2 ng/ml. Each protein dilution was detected thrice by an enzyme linked immunosorbent assay (ELISA) (Wantai, Beijing, China). A standard curve was drawn, based on the concentration and OD value. The linear range was from 3 ng/ml to 30 ng/ml with R^2^ = 0.999. The serial gradient fractions were denatured and the quantity of ORF2 protein was determined. HEV RNA was quantified using real-time RT-PCR. HEV RNA extraction was performed using a Total RNA Kit (QIAGEN, Dusseldorf, Germany) and the extracted RNA was subjected to real-time RT-PCR with an HEV RNA real-time RT-PCR kit (Wantai, Beijing, China). To target the ORF2 and ORF3 overlapping region, sense and antisense primers and a probe with a 5′ reporter dye (FAM) and a 3′ quencher dye (TAMRA) were used in a real-time RT-PCR assay as described previously[17]. Amplification was at 50°C for 30 min, 5 cycles of 95°C for 15 sec, 50°C for 30 sec and 72°C for 1 min, then 40 cycles of 95°C for 15 sec and 55°C for 40 sec. The diluted standard HEV RNA of 7×10^4^ copies/μL to 7×10^7^ copies/μL was detected thrice. The standard curve was drawn based on concentration and Ct value. The accuracy of the real time RT-PCR for quantification of HEV RNA was validated with a 10 fold serial dilution series of HEV RNA. Linear range was from 7×10^4^ copies/μL to 7×10^7^ copies/μL with R^2^ = 0.999. The RNA content was determined in serial gradient fractions.

#### 3. Equilibrium centrifugation in a sucrose density gradient

A density gradient was prepared in CP70ME tubes (Hitachi, Tokyo, Japan), [1 ml each of 60%, 40%, and 30%, 2 ml of 20% and 10% (w/w) sucrose in TE buffer supplemented with 150 mM NaCl)]. Three milliliters of fecal suspension or culture supernatant from infected PLC/PRF/5 cells were treated with 20μg/ml of RNase A at 37°C for 1 hour and layered onto the gradient. The tubes were centrifuged at 35000 rpm at 4°C for 4 h. Seven fractions of 1 ml and seventeen fractions of 200 μl were recovered from the top. The density of each fraction was measured by refractometry.

#### 4. The removal of lipid and ORF3 from the virus surface

The removal of lipid: The ultracentrifugation fractions rich in HEV RNA from the two sources were incubated with 2% NP40 at 37°C for 1 h. Then, buffer containing 2% NP40 was replaced with PBS buffer. The delipidated samples were ultra-centrifuged again and the fractions were harvested as above.

The removal of ORF3 protein: The delipidated samples were treated with a solution containing 0.1% (vol/vol) NP40, 0.1% (vol/vol) 2-mercaptoeththanol (2-ME) and 0.1% (wt/vol) pronase E and then was incubated at 37°C for 2 h. The above solution was replaced with PBS buffer using a centrifugal filter (100K, Millipore, Merck, Germany). The samples were ultra- centrifuged again and the RNA rich fractions were harvested as above.

#### 5. Infectivity assay

PLC/PRF/5 cells were used to evaluate the infectivity of the gradient fractions. Monolayers of PLC/PRF/5 cells in six-well microplates were incubated with 0.1ml gradient fractions and 3ml MEM with 2% FBS. Three days after inoculation, the solution was removed and 3 ml MEM with 2% FBS was added. The culture was incubated at 35°C in a humidified 5% CO_2_ atmosphere. Then, every three days, the maintenance medium was collected and stored at -80°C until virus titrations were performed.

#### 6. Virus binding assay

PLC/PRF/5 cells grown in 12-well plates were incubated with the samples with the same content of HEV RNA. After incubation at 37°C for 8h, 24h, 48h and 72h, the cells were washed with PBS five times. Then the RNA binding to the cells was extracted and the amount was measured using RT-PCR quantification analysis, as described above.

#### 7. Purification of HEV ORF2 protein and HEV

The ORF2 protein and HEV were extracted using affinity chromatography. The anti-ORF2 monoclonal antibody (the antibody was produced in NICPBP lab[18]) was purified using rProtein G FF (GE Healthcare) and coupled to CNBr-activated Sepharose 4B (GE Healthcare, Fairfield, CT) at 5mg per ml gel. The gradient fraction rich in ORF2 protein was incubated with the coupled Sepharose 4B and rotated for 1 hour at room temperature. After the samples were loaded with beads into the tube, five gel volumes of PBS were used to remove impurities. Then, the ORF2 protein was eluted with elution buffer (0.1M sodium acetate, 0.5M NaCl, pH 4.5) in two gel volumes and was concentrated using a centrifugal filter (10K, Millipore, Merck, German).

The anti-ORF3 monoclonal antibody (the antibody was produced in NICPBP lab and the data was unpublished) was purified and coupled to the CNBr-activated Sepharose 4B (GE Healthcare) as described previously. The gradient fraction rich in HEV RNA was incubated with 2% NP40 at 37°C for 1 h. Then the buffer containing 2% NP40 was replaced with PBS. The treated fraction samples were incubated with the coupled Sepharose 4B in PBS buffer with rotation overnight at 4°C. After the samples were loaded, five gel volumes PBS were used to remove impurities. The virus was eluted with elution buffer (0.1M sodium acetate, 0.5M NaCl, pH 4.5) in two gel volumes and then concentrated using a centrifugal filter (3 K, Millipore, Merck, Germany).

#### 8. Immunocapture RT-PCR assay using anti-ORF3

Immunocapture RT-PCR was used to evaluate the ability of virus from various sources to bind anti-ORF3 antibody. The samples with or without delipidation were bound to anti ORF3 MAb beads as described above and the beads were eluted with lysis buffer (100 mM Tris-HCl, pH 7 5, 500 mM LiCl, 0 5% LiDS,1 mM EDTA, 5 mM DTT). RNA was detected in the eluate using real-time RT-PCR. The binding RNA percentage was calculated as the bound RNA divided by the total RNA in the original sample.

#### 9. Western blotting, glycoprotein staining and protein analysis using LC-MS/MS

The samples were resolved by sodium dodecyl sulfate-polyacrylamide gel electrophoresis (SDS-PAGE) at a 15% concentration and electrophoretically transferred onto a polvinylidene difluoride membrane (PVDF) (0.2μm; GE Healthcare) for analysis by western blotting. The membrane was incubated with serum from a hepatitis E patient (for ORF2 protein) or with rabbit anti-ORF3 antibody for 1 hour at room temperature. Then the membrane was incubated with alkaline phosphatase conjugated secondary antibody (1:10,000) (ZSGB-BIO, Beijing, China) and, after washing, bands were detected with BICP/NBT (MP, Santa Ana, CA).

Glycoprotein staining assay: The ORF2 protein extracted from the cell culture supernatant was separated by SDS-PAGE at a 15% concentration and then the gel was stained using a Glycoprotein Staining Kit (Thermo Scientific, Waltham, MA).

The ORF2 protein captured from cell culture supernatants was separated by SDS-PAGE. Then the positive band was excised from the region of the gel corresponding to the western blot and hydrolyzed overnight with trypsin treated with N-tosyl-l-phenylalanine chloromethyl ketone (Sigma-Aldrich, St. Louis, MO.) at 37°C.The digested samples were initially transferred to the pre-column at a flow rate of 5 μl/min with mobile phase A (0.1% formic acid). Then the peptides were separated with a linear gradient of 10–40% mobile phase B (0.1% formic acid in acetonitrile) over 60 min at 200 nl/min followed by 10 min at 85% mobile phase B. The column temperature was maintained at 35°C. Analysis of tryptic peptides was performed using a SYNAPT G2-S mass spectrometer (Waters, Milford, MA, USA) operating in the v-mode with a typical resolving power of at least 10,000 full-width half-maximum. The TOF analyzer was calibrated with the MS/MS fragments of [Glu1]-fibrinopeptide B from m/z of 50 to 1600. Accurate mass LC-MS and MS/MS data were collected in high-definition DDA mode. LC-MS/MS data were processed using ProteinLynx Global Server version 2.4 and the resulting peak lists were searched against the sequence of genotype 4 ORF2 protein (HEV AJ272108).

#### 10. The refolding of ORF2 protein and RNA

Reassembly of the captured ORF2 protein and RNA was performed according to the procedure described by Touze et al.[19]. Briefly, 550ng captured ORF2 protein was incubated in 50mMTris–HCl buffer (pH 7.5)containing 150 mM NaCl, 1 mM EGTA and 20 mM DTT in a final volume of 500 μl at room temperature for 1 hour.100μg HEV RNA in 50 mM Tris–HCl (pH 7.5),150 mM NaCl and 1% DMSO was then added to the protein. The mixture was dialyzed against 50 mM Tris–HCl (pH 7.5) containing150 mM NaCl and 5mM CaCl_2_ at 4°C overnight and then mixture treated with 20μg/ml RNase A (Sigma-Aldrich, St. Louis, MO) at 37°C for 1 hour and ultracentrifuged as described previously. The HEV RNA, with or without treatment with RNase A, as the RNA negative control and the ORF2 protein without HEV RNA as the protein control were also ultracentrifuged using the same protocol. The RNA and the ORF2 protein in the fractions were detected using real-time RT-PCR and ELISA.

To observe the RNA content variation with the added ORF2 protein, ORF2 protein was diluted serially to 5500 ng/ml, 550 ng/ml, 55 ng/ml, and 5.5 ng/ml and was mixed with 100μg HEV RNA. Then the processing method was as described previously. The RNA was detected in the various fractions and compared with native virus.

#### 11. Electron microscopy

Electron microscopy was performed on a Hitachi H-7500 electron microscope (Hitachi, Tokyo, Japan), at 80 kV. The refolded samples banded at a density of 1.19–1.24 g/cm^3^were stained with 2% uranyl acetate and observed by electron microscopy.

#### 12. Electrophoretic Mobility Shift Assay (EMSA)

The full-length HEV cDNA was reversed transcripted from genotype 4 HEV RNA [16]and was cloned into pcDNA3.1 vector(Invitrogen, Carlsbad, CA). Then it was linearized with MluⅠ. RNAs were synthesized by transcribing linearized plasmid DNAs using transcription kit (Thermo Scientific, Waltham, MA) at 37°C for 2 hours. The synthesized RNA was extracted with phenol/chloroform, and then precipitated with ethanol. The purified RNA 3’ end was labeled with desthiobiotin using T4 RNA ligase (Thermo Scientific).

To detect RNA-protein interactions by EMSA, the captured ORF2 protein were mixed with 5 pmol biotin-labeled RNA and incubate for 30 min in 100mM HEPES, 200mM KCl, 10mM MgCl_2_, 10mM DTT, pH 7.3 in 20 μl on ice. Specific competitive inhibition EMSA was using 4 times and 8 times unlabeled HEV RNA as competitor and nonspecific competitive inhibition EMSA was using 0.2μg total PLC/PRF/5 cell RNA as competitor. The competitor non-biotin-labeled RNA was mixed well with biotin-labeled RNA and then was mixed with binding protein, the mixture was incubated in binding buffer on ice. The mixtures were analyzed on native 0.4% agarose gels using Tris-acetate-EDTA buffer. The samples were electrophoresed until the bromophenol blue dye has migrated approximately 3/4 down the length of the gel. Sandwich the gel and the soaked nylon membrane (Hybond, GE,Fairfield, OR) in a clean electrophoretic transfer at 400mA for 30 minutes. Then the membrane was cross-linked using UV-light 5 min and was blocking. The membrane was incubated with streptavidin-horseradish peroxidase conjugate buffer. After washing, the labeled RNA was detected by chemiluminescence.

## Results

To analyze the hepatitis E virions in the cell culture supernatant and in feces, we ultra-centrifuged the virus from the two sources and analyzed the various fractions rich in RNA or ORF2 protein.

### 1. The HEV RNA and HEV ORF2 proteinbandedat different densities during ultracentrifugation

**Fig 1:**
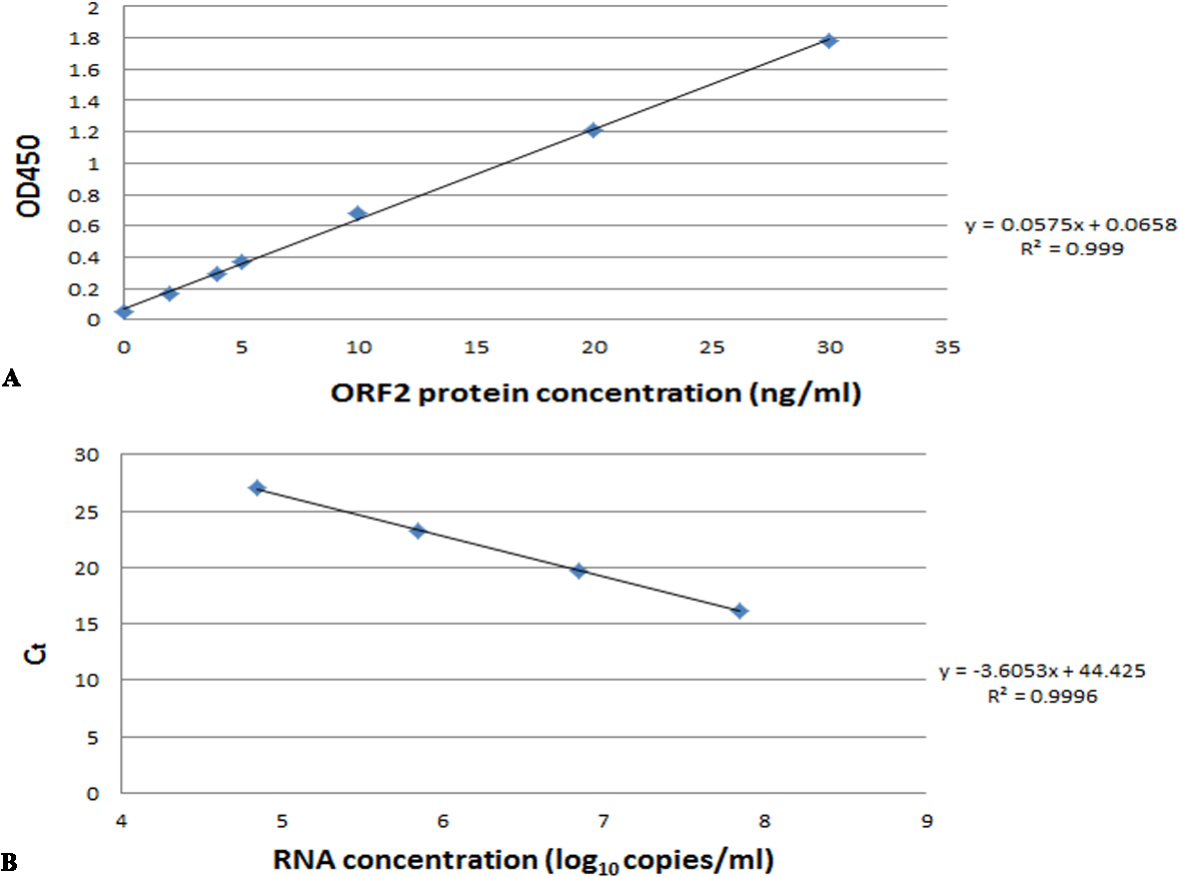
The standard curve of ORF2protein (A) and HEV RNA (B). (A): The X-axis is the concentration of ORF2 protein standard and Y-axis is the value of OD_450_. (B): X-axis is the concentration of HEV RNA standard and Y-axis is the Ct value of real time RT-PCR.

The accuracies of the ELISA for quantification of ORF2 protein and the real time RT-PCR for quantification of HEV RNA were validated. The linear ranges are shown in Fig 1A and 1B.

To extract and analyze the hepatitis E virion, virus preparations from the cell culture supernatant and feces with similar RNA contents were separated using sucrose density gradient ultracentrifugation (10%-60% (wt/vol)) and the gradient fractions were harvested. The ORF2 protein and HEV RNA in the fractions were analyzed using ELISA assay and real-time RT-PCR. Detection of HEV RNA in the gradient of the fecal preparation shows a single band with a peak in fraction 21. Although there was little ORF2 protein in this band, the density of 1.21 g/cm^3^ is consistent with this containing the hepatitis E virions. Most of the detectable ORF2 protein remained at the top of the gradient; very little RNA was detected in these fractions and it seems possible that this is free capsid protein, perhaps derived from disrupted virions in the feces. A similar pattern was seen in the gradient of the virus from cell culture. However, the peak of the major band of HEV RNA was in fraction 15, with a density of 1.15 g/cm^3^. Again, most of the ORF2 protein remained at the top of the gradient and this may be free protein secreted from the infected cells. In addition, there was a second band of HEV RNA with a peak in fractions 5 and 6, with a density of 1.05–1.06 g/cm^3^.

To test whether the ultracentrifugation conditions might disrupt the hepatitis E virions, the samples from both cell culture supernatant and feces were fixed using 4% polyformaldehyde and then ultracentrifuged under the same conditions. The results did not change: the ORF2 protein and RNA distribution were the same as previously. So the structure of HEV was stable under the ultracentrifugation conditions.

### 2. HEV infectivity was associated with the fractions rich in HEV RNA

The ORF2 protein and RNA were distributed in different fractions following the ultracentrifugation. Culture in PLC/PRF/5 cells was used to investigate the infectivity of gradient fractions of HEV from the cell culture supernatant and feces.

**Fig 2:**
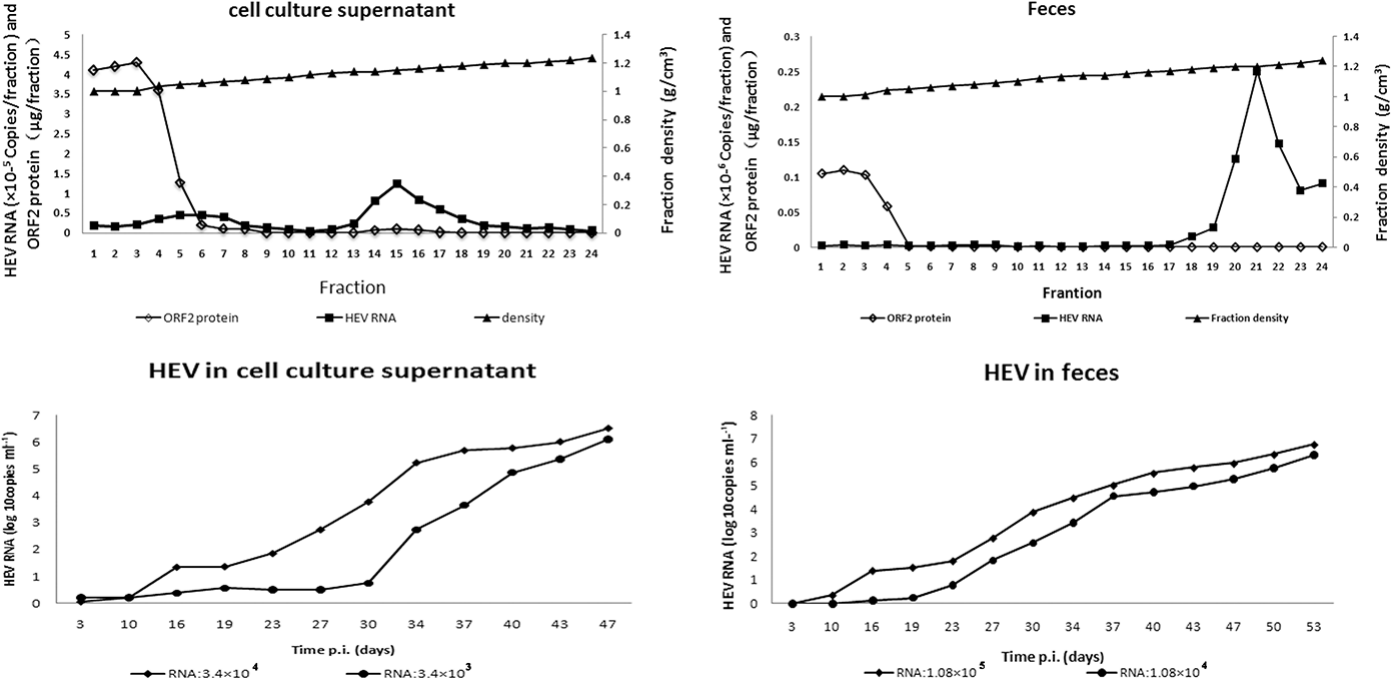
A and B are density-gradient fraction of HEV in cell culture supernatant (A) and feces (B). The X-axis shows the fraction number, the left Y-axis is the ORF2 protein content (μg/ fraction) and the RNA content (10^5^copies/fraction of cell culture supernatant HEV and 10^6^ copies/fraction of feces HEV) in the fraction and the right Y-axis is the density of the fraction. C and D are quantification of HEV RNA in culture supernatant of PLC/PRF/5 cells incubated with virus from cell culture (C) and from feces (D). The X-axis is the time post incubation and the Y-axis is the HEV RNA concentration.

The infection and replication ability of fraction 15 from the cell culture supernatant is shown in Fig 2C. The progeny HEV titer reached 5×10^2^ copies/ml at 27 days and 34 days post inoculation (pi) when the titer of inoculated HEV RNA was 3.4×10^3^copies/ml and 3.4×10^4^copies/ml, respectively. The progeny HEV titer reached 1×10^6^ copies/ml at 43 days and 47 days. HEV continued to be released into the cell supernatant, reaching a peak at 60 days post incubation. The second band of HEV RNA from culture cell supernatant, which was at fraction 5 with a density of 1.06g/cm^3^, was also tested for infection and replication ability. No progeny HEV was detected in the culture cells within an observation period of two months. Because the sample from the cell culture supernatant had been treated with 20μg/ml of RNase A before ultracentrifugation, the HEV RNA at the second band was assumed to be protected from digestion of RNase A. Thus, although the HEV RNA in this fraction seemed to be protected the particles were not infectious in cell culture.

The infection and replicative ability of fraction 21 from the sample from feces is shown in Fig 2D. The progeny HEV titer reached 5×10^2^ copies/ml at 27 days and 34 days post inoculation (pi) and reached 1×10^6^ copies/ml at 50 days and 53 days, when the titer of inoculated HEV RNA was 1.08×10^5^ copies/ml and 1.08×10^4^ copies/ml, respectively. HEV continued to be released into the cell supernatant, reaching a peak at 63 days post infection. The curve of progeny HEV titer and time post inoculation implies that virus production varied with the titer of HEV inoculated.

### 3. ORF3 protein and lipid co-existed on the virus surface in the RNA rich fraction from cell culture but not that from feces; the lipid has no influence on virion binding to the cell but the ORF3 protein interferes with binding

**Fig 3:**
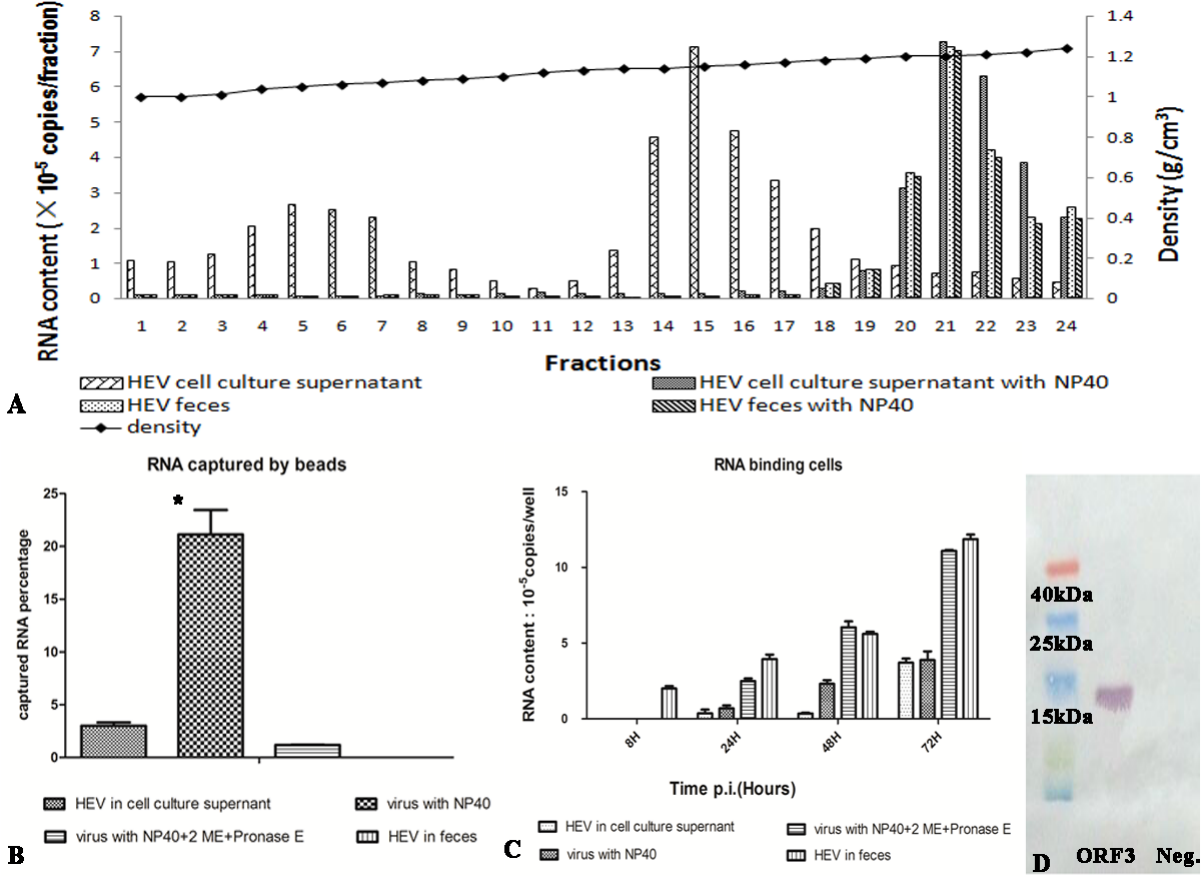
The analysis of the fraction rich inHEV RNA. (A) The density-gradient fraction of virus from different sources and with different treatments. The X-axis is the fraction number and the right Y-axis is the density of the fraction. The left Y-axis is the HEV RNA content. (B) The binding ability of virus to anti-ORF3 MAb, the Y-axis is the percentage of binding HEV RNA to total HEV RNA of sample. The * represents the P value of HEV virus in the cell culture supernatant with NP40 treatment compared to the same sample without NP40 treatment. P value is less than 0.05. (C) The binding effect of virus to cells, the X-axis is the time post incubation with samples and the Y-axis was the binding RNA content to cells. (D) The fraction 15 of cell culture supernatant was detected by western blotting with an anti-ORF3 MAb.

The RNA-rich fractions of virus from the cell culture supernatant and from feces were both of a higher density than the ORF2-rich fractions. However, they were not of the same density (Fig 3A); the RNA-rich particles from feces had a density of 1.21g/cm^3^ whilst those from the culture supernatant were of 1.15g/cm^3^. After treatment of the samples with NP40 to remove lipid, the density of the particles from the cell culture supernatant changed from 1.15 g/cm^3^ to 1.21 g/cm^3^ but the density of those from feces did not change (Fig 3A). This suggests that the virus in the cell culture supernatant was enveloped with lipid and banded at a lower density.

The distribution of ORF3 protein was investigated using immunocapture RT-PCR assay. Virions from the cell culture supernatant, virions treated with NP40 and virions treated with NP40, 2-ME and pronase E, were mixed with the beads coupled with anti-ORF3 MAbs. Then the percentage of immunocaptured HEV RNA was determined (Fig 3B). 3% of virions from the cell culture were captured by the beads and this increased to 20% after treatment with NP40. This then decreased to 1.2% after treatment with NP40, 2-ME and pronase E. This suggested that the ORF3 protein on the virus surface was covered in lipid and there was little ORF3 protein on the lipid surface. However, the percentage of RNA captured from the virions from the feces was 0.3%, with and without treatment with NP40.This was indicates there was very little ORF3 protein on the surface of the virions from feces.

The virions, with or without these treatments were incubated with cells to analyze the influence of lipid and ORF3 protein on binding to the cells (Fig 3C). The virions from feces and the virions without ORF3 protein show the strongest binding. The binding RNA of virions from feces and without ORF3 was 3.96×10^5^ copies and 2.50×10^5^ copies per well, respectively, at 24 h incubation, and 1.86×10^6^ copies and 1.09×10^6^copies per well, respectively, at 72 h incubation. The binding of the two preparations was similar; the binding capacity of the virions before and after delipidation also was similar but was lower than the virions from feces and those without ORF3 protein. The binding of RNA was 3.62×10^4^ copies and 6.82×10^4^copies per well before and after delipidation, respectively, at 24 h incubation, and was 3.71×10^5^ copies and 3.87×10^5^ copies per well at 72 h incubation. The binding capacity of the virions without ORF3 protein was about three times that of the virions with ORF3 protein. This implies that the lipid on the surface has no influence on the binding of the virions to cells but the ORF3 protein interferes with binding.

To confirm the presence of the ORF2 and ORF3 proteins, the RNA-rich fractions from cell culture and feces were probed with anti-ORF2 and-ORF3 antibodies in western blot assays. Only the virus from cell culture showed a clear band of ORF3 protein (Fig 3D). ORF3 protein was not detected in the virus from feces and ORF2 protein could not be detected in either sample, perhaps due to the low amount of ORF2 protein in the RNA-rich fractions.

No ORF2 was detected in the RNA-rich fractions from cell culture or feces using a commercial ELISA. Because the binding to ORF3 antibody increased significantly after NP40 treatment of the virus from cell culture, the treated fraction was concentrated using beads coupled with anti-ORF3 MAb. The RNA and ORF2 protein content of the samples were 7.68×10^8^ copies/ml and 8.25 ng/ml, respectively, after tenfold concentration. This implied that the RNA and ORF2 and ORF3 proteins were in one structure in the material derived from cell culture. The absence of the ORF3 protein from the surface of the virus from feces precluded an equivalent analysis of that virus. We tried to compare the ratio of RNA to ORF2 protein between the virus from feces and the virus captured by anti-ORF3 MAbs. However, the ratio of virus from fecesvaried when the samples came from different patients. The relationship between them is still under study.

### 4. The ORF2 protein present in the low density fractions is a glycoprotein and binds HEV RNA to form particles which band at high density

Because the ORF2 protein present in the RNA-rich fraction was not detectable by western blotting, we captured and analyzed the ORF2 protein in the low density fractions from the cell culture supernatant. The quantity of ORF2 protein in the preparation from the feces was too low to detect.

**Fig 4:**
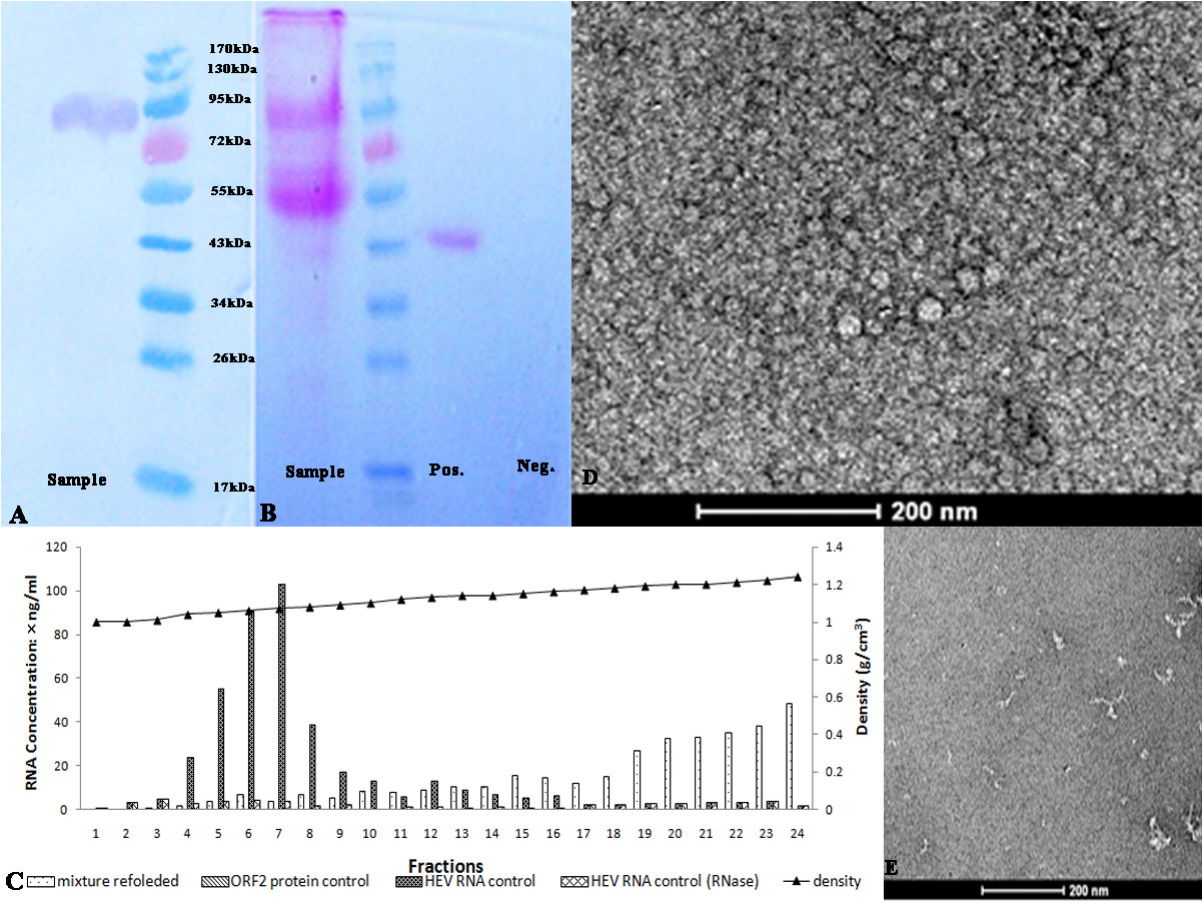
Analysis of the ORF2 protein from the cell culture supernatant. (A) Western blotting of the protein using an anti-ORF2 MAb. (B) The glycoprotein staining of the SDS-PAGE gel. The sample containsthe same magenta band as the positive control and the negative control has no band. (C) The RNA content in density-gradient fractions of the RNA-ORF2 binding product and controls. The X-axis is the fraction number, the left Y-axis is the HEV RNA content andthe right Y-axis is the density of the fraction.(D) and (E) Transmission electron microscopy of the RNA-ORF2 binding product (D) and ORF2 protein without HEV RNA(E) stained with uranyl acetate.The scale bar indicates 200 nm.

The ORF2 protein in the low density fractions was extracted using affinity chromatography with anti-ORF2 MAbs and was analyzed. An 88kDa band was detected by western blotting using anti-ORF2 MAbs (Fig 4A). This band stained positive using the periodic acid-Schiff method and was confirmed to be a glycoprotein (Fig 4B).The corresponding region ofthe SDS-PAGE gel was excised and analyzed using LC-MS/MS. The 14 fragments identified were consistent with the HEV ORF2 sequence and distributed in the region 101aa to 643 aa of the ORF2 protein. These results indicated that the protein captured using affinity chromatography was the HEV ORF2 protein.

Since RNA binding is the extrinsic factor essential for the assembly of HEV native capsid[20]. To test whether the captured ORF2 protein could bind to and protect HEV RNA, we mixed RNA and ORF2 protein in the buffer containing 5mM CaCl_2_, then the refolded mixture was separated using ultracentrifugation. The relationship of the quantity of the product with the amount of ORF2 protein added was determined. When the RNA content was 100μg, the RNA content in RNA-ORF2 binding product was 0.05μg, 0.37μg and 3.55μg when 5.5 ng, 55 ng and 550 ng ORF2 protein was added. However, when the amount of ORF2 protein was increased to 5500ng, the RNA content of RNA-ORF2 binding product decreased to 3.32μg.

The ORF2 protein, HEV RNA and the binding product of ORF2 protein and RNA were ultracentrifuged and the RNA and ORF2 protein distribution were analyzed. The RNA distribution was shown in Fig 4C. The ORF2 protein banded at the density of 1.00 g/cm^3^ to 1.04 g/cm^3^ after ultracentrifugation of 550ng ORF2 protein (S2 Fig), whilst the RNA banded at the density of 1.04g/cm^3^ to 1.08 g/cm^3^ after ultracentrifugation and the amount of RNA decreased after incubation with RNase A. The distribution of ORF2 protein did not change but the density of the RNA–rich band changed to 1.19g/cm^3^ to 1.24 g/cm^3^ after ultracentrifugation of the binding product of RNA and ORF2 protein. The RNA content in this fraction did not change when the RNA-ORF2 binding product was incubated with RNase A. The amount of RNA in the RNA-ORF2 binding product varied with the amount of ORF2 protein added, the percentage of ORF2 protein in this fraction was 0.03% of all added ORF2 protein. The virus from feces also banded at this density. These results suggest that the ORF2 protein can bind to and protect the RNA and the density of some product was similar to that of the virus from feces.

We observed using electron microscopy the RNA-ORF2 binding product which banded at the density of 1.19–1.24g/cm^3^ (Fig 4D). Several particles may be seen with a size of about 37 nm.

### 5. Specific Competitive Inhibition Electrophoretic Mobility Shift Assay (EMSA)

**Fig 5:**
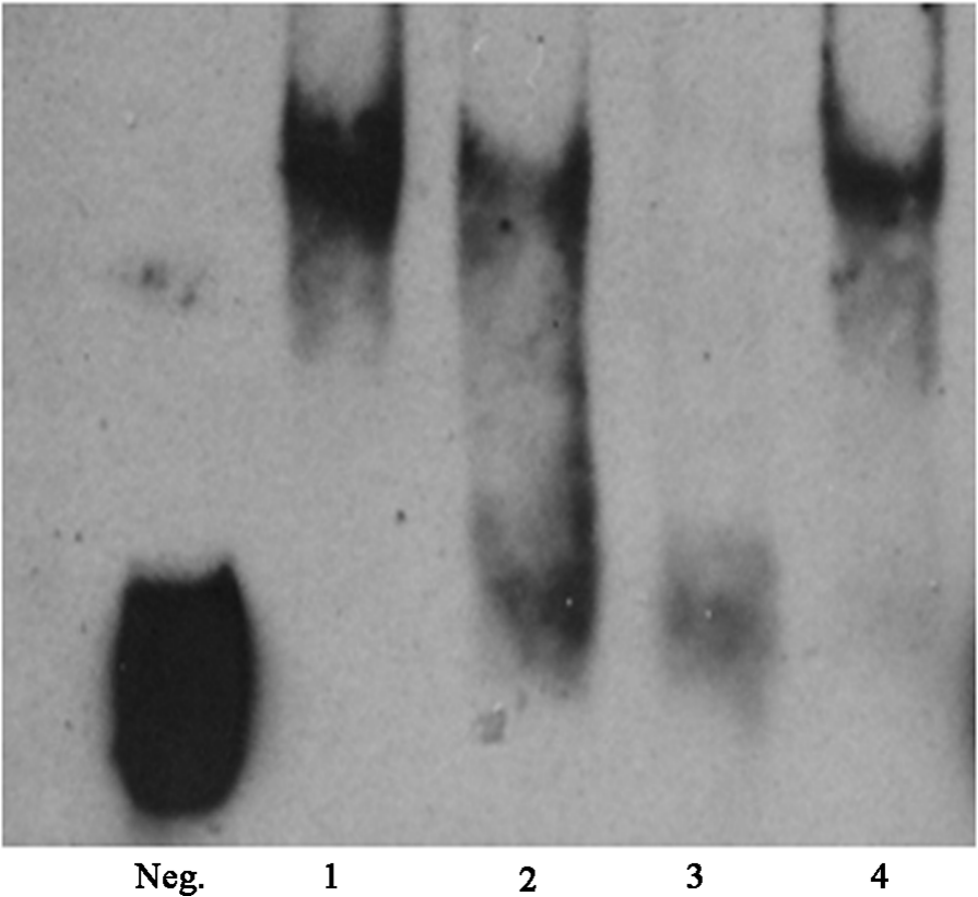
Specific Competitive Inhibition EMSA of HEV RNA and captured ORF2 protein. The biotin-RNA without ORF2 protein was as negative control. The assay was carried out as described under “Materials and Methods”. The non-biotin labeled RNA in lane 2 and 3 were 4 fold and 8 fold to biotin labeled RNA. The lane 4 was shown the nonspecific competitive inhibition of 0.2μg total PLC/PRF/5 cell RNA.

We carried out a competitive inhibition electrophoretic mobility shift assay to elucidate the binding specificity of captured ORF2 and HEV RNA. Fig 5 shows the HEV RNA binding activity of captured ORF2 protein as a function of increasing amount of non-biotin-labeled HEV RNA as a competitor. The 80% competition was observed in the presence of 4-fold non-biotin-labeled HEV RNA, and the binding activity of the biotin-RNA with the captured ORF2 protein was replaced completely with non- biotin-RNA in the presence of 8-fold non-biotin-labeled HEV RNA. The biotin-RNAs without binding proteins were partially degraded with the enzymes produced by lysed cells. The total RNA of PLC/PRF/5 does not affect the binding action because of the lack of specific binding sites.

## Discussion

In the absence of a productive cell culture system, molecular studies of hepatitis E virus were based on two approaches: the transfection of infectious HEV cDNA clones and the expression of individual viral proteins. Little information has been gained by studying the native virus. In this study, the composition of virus produced in a recently established cell culture system was analyzed and compared with virus excreted from the feces of acutely infected monkeys.

Analysis of the ultracentrifugation result showed that most ORF2 protein was separate from the HEV RNA; the infectivity was associated with the HEV RNA and the excess of ORF2 protein was not in particulate form and had no replicative capacity. Virions from the two sources, feces and cell culture supernatant, formed single bands, which were infectious but of different density. The density of the virions from the cell culture supernatant changed to that of virions from feces when the lipid was removed with NP40. This indicates that, like hepatitis A virus (HAV), HEV from cultured cells can hijack the host membrane to form an envelope.

However, there is a difference between the viruses. HAV from cell culture system forms two RNA-rich fractions following the ultracentrifugation; a proportion of HAV hijacks the host membrane to bud from cell and the remainder is secreted without the membrane. The latter has no envelope and bands at a high density following ultracentrifugation. However, HEV virions from the cell culture supernatant formed only one band after ultracentrifugation; this was at low density and was shown to be enveloped with lipid. It seems that naked hepatitis E virions are not released from infected PLC/PRF/5 cells in detectable amounts.

HAV and HEV are similar; both form naked virions that are spread via the fecal-oral route but also form enveloped particles that circulate in the plasma of the infected host [21]. It is not known whether the lipid envelope contains viral proteins. Our study found that only 3% of virions bound to anti-ORF3 MAbs when the virion were not treated with detergent but the binding percentage increased to 20% when the virions were delipidated. This result is similar to other studies [7, 14], indicating that there is little ORF3 in the envelope and most of the ORF3 protein is protected by lipid.The study of Takahashi papered al.[8] showed that anti-ORF3 MAbs can capture the HEV particles but their later study [14] shows that the same Mabs immunocapture the virions more effectively after delipidation of the samples.

Investigation of the ability of the virions, with and without detergent treatment, to bind to cells suggests that the envelope has little effect on cell binding. However, removal of the ORF3 protein increased the cell binding ability greatly, suggesting that the ORF3 protein has a negative effect on binding. This may support the hypothesis thatthe ORF3 protein only functions to facilitate the egress the virus from the infected cells [5] and is not involved in cell to cell spread. This accords with the absence of the ORF3 protein from the virus from feces; clearly, the protein is not required for the transmission of the virus and infection of a new host.

The analysis of ORF2 by LC-MS/MS revealed that a peptide in the C-terminal 52 amino acids of the protein was present in the native ORF2 protein. This result is consistent with the study of Shiota et al.[22]. The HEV vaccines produced by Glaxo Smith Kline and Xiamen Innovax Biotech do not contain the C-terminal 52 amino acids. The studies of Field and Wang and their co-workers[23, 24] revealed that several peptides in the C-terminal 573–660 aa region containstrong IgG and IgM antigenic epitopes. The peptides from the C-terminal 600 amino acids have stronger reactions. Including the C-terminal 52 amino acids of ORF2 in vaccine formulations may enhance the production of neutralizing antibodies.

The analysis of ORF2 protein by western blotting revealed an apparent molecular weight of 88 kDa, similar to that produced in mammalian cells but differing from the findings of Emerson et al.[25] and Shiota et al.[22] who reported an apparent molecular weight of 75 kDa. LC-MS/MS analysis confirmed that the extracted protein was the ORF2 protein. However, the ORF2 protein assembled into the virus was not detectable because of the low amount of virus produced by the HEV culture system and it is not known whether the ORF2 protein assembled into the virus is glycosylated. We refolded the ORF2 protein and RNA in the buffer used to assemble HPV [19] to determine whether the glycosylated ORF2 protein can bind RNA and form a stable structure. We also determined the specific packaging of captured ORF2 and HEV RNA using specific competitive inhibition electrophoretic mobility shift assay. The result was means that the ORF2 protein can bind HEV RNA specifically. Some refolded structure has a similar sedimentation coefficient to the native virus in feces and the distribution of ORF2 protein and RNA was also similar to the native virus. This indicates that the glycosylated ORF2 protein, which remains on top of the gradient, can bind to and protect HEV RNA.

Because no efficient cell culture was available, the natural form of ORF2 protein in the HEV virion, including its length and glycosylation status, is not well known. Jameel et al.[10, 26]detected the ORF2 protein expressed using a mammalian cell system and showed the protein was glycosylated. Torresi et al.[27] also studied the ORF2 form in the cytosol and membrane in a similar system; the result suggested that glycosylated ORF2 protein may not be an intermediate in HEV capsid assembly and may be an artificial product of heterologous expression.Graff’s study [28] using infectious cDNA clone showed that the majority of ORF2 protein in cells is not glycosylated. Although the HEV virion cannot be produced if the three glycosylated sites are disrupted by mutation, it was thought that the failure of HEV virion production was caused by perturbing the structure rather than the elimination of glycosylation. So far, no studies have confirmed the natural form of ORF2 protein in the virion. Although the ORF2 structure was not analyzed in the present study, it was confirmed that the ORF2 protein from cell culture was mostly glycosylated and can bind HEV RNA. This suggested that the glycosylated ORF2 may be the natural form in HEV virion.

The non-glycosylated ORF2 protein, without the C-terminal 52 amino acids, can assemble into particles with a T = 1 structure and the protein will assemble into particles with a T = 3 structure in the presence of RNA. One study [20] suggested that RNA binding is an intrinsic factor essential for the assembly of the hepatitis E virion. This was also reflected in our study. Most of the ORF2 protein was in a non-particulate form without the RNA and a portion of this ORF2 protein changed in the presence of RNA, forming a structure with a similar sedimentation coefficient to the native virus in feces.

In conclusion, the ORF2 protein and the viral RNA banded at different density following ultracentrifugation. ORF3 protein and lipid was on the surface of the virus from the cell culture supernatant but not the virus from feces. The glycosylated ORF2 protein containing the C-terminal 52 amino acids in the low density fractions of the gradient can bind to and protect the viral RNA, suggesting that this may be the protein assembled into virus produced in cell culture.

## Supporting Information

S1 Fig The binding effect of virus to cells, the X-axis is the time post incubation with samples and the Y-axis was the binding RNA content to cells. (TIF)

S2 Fig The ORF2 protein content distribution of the ORF2 negtive control and the mixture. (TIF)

S1 Table The specific packaging of proteins to HEV RNAandsendai virus RNA. (XLSX)
