# Supplementary figures and images for: Hepatitis E Virus Produced from Cell Culture Has a Lipid Envelope
Source: PLoS One. 2015 Jul 10;10(7):e0132503. doi: 10.1371/journal.pone.0132503 (PMC4498737; doi:10.1371/journal.pone.0132503)

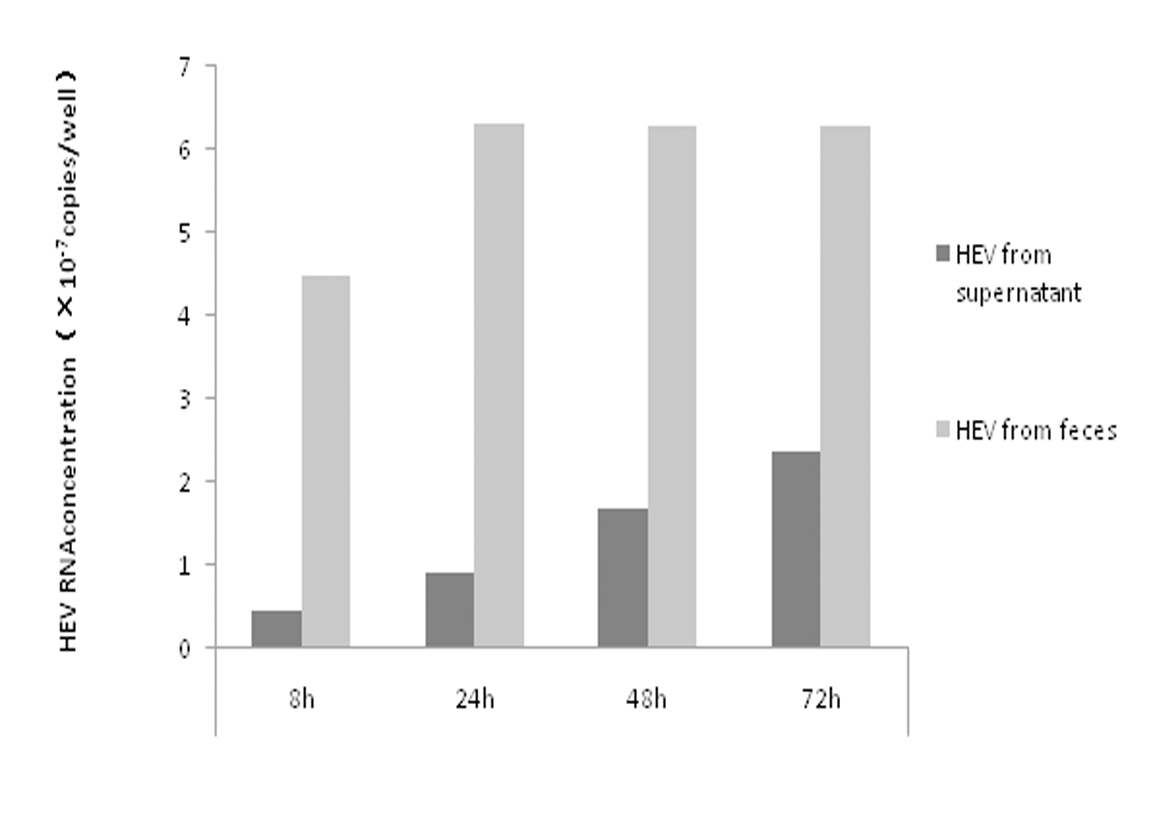

Supplement: S1 Fig — (TIF) [file pone.0132503.s001.tif]

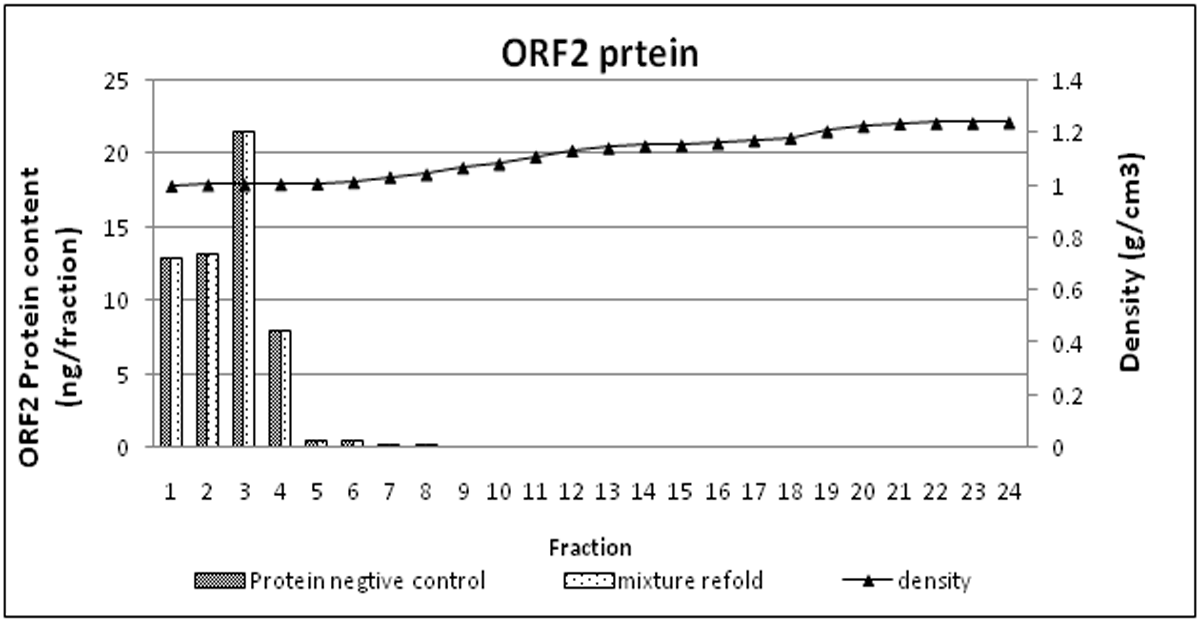

Supplement: S2 Fig — (TIF) [file pone.0132503.s002.tif]
